# *In situ* serial crystallography facilitates 96-well plate structural analysis at low symmetry

**DOI:** 10.1107/S2052252524005785

**Published:** 2024-07-15

**Authors:** Nicolas Foos, Jean-Baptise Florial, Mathias Eymery, Jeremy Sinoir, Franck Felisaz, Marcus Oscarsson, Antonia Beteva, Matthew W. Bowler, Didier Nurizzo, Gergely Papp, Montserrat Soler-Lopez, Max Nanao, Shibom Basu, Andrew A. McCarthy

**Affiliations:** a European Molecular Biology Laboratory Grenoble Outstation, 71 Avenue des Martyrs 38042Grenoble France; b European Synchrotron Radiation Facility 71 Avenue des Martyrs 38042Grenoble France; Chinese Academy of Sciences, China

**Keywords:** *in situ* serial crystallography, room-temperature structures, crystallization plates, conformational flexibility, plate holders, triclinic symmetry, macromolecular crystallography, autotaxin

## Abstract

The advent of serial crystallography has rejuvenated and popularized room-temperature X-ray crystal structure determination. Structures determined at physiological temperature reveal protein flexibility and dynamics. In addition, challenging samples (*e.g.* large complexes, membrane proteins and viruses) form fragile crystals that are often difficult to harvest for cryo-crystallography. Moreover, a typical serial crystallography experiment requires a large number of microcrystals, mainly achievable through batch crystallization. Many medically relevant samples are expressed in mammalian cell lines, producing a meager quantity of protein that is incompatible with batch crystallization. This can limit the scope of serial crystallography approaches. Direct *in situ* data collection from a 96-well crystallization plate enables not only the identification of the best diffracting crystallization condition but also the possibility for structure determination under ambient conditions. Here, we describe an *in situ* serial crystallography (iSX) approach, facilitating direct measurement from crystallization plates mounted on a rapidly exchangeable universal plate holder deployed at a microfocus beamline, ID23-2, at the European Synchrotron Radiation Facility. We applied our iSX approach on a challenging project, autotaxin, a therapeutic target expressed in a stable human cell line, to determine the structure in the lowest-symmetry *P*1 space group at 3.0 Å resolution. Our *in situ* data collection strategy provided a complete dataset for structure determination while screening various crystallization conditions. Our data analysis reveals that the iSX approach is highly efficient at a microfocus beamline, improving throughput and demonstrating how crystallization plates can be routinely used as an alternative method of presenting samples for serial crystallography experiments at synchrotrons.

## Abstract 2

The determination of a challenging structure in the *P*1 space group, the lowest symmetry possible, shows how our *in situ* serial crystallography approach expands the application of crystallization plates as a robust sample delivery method.

## 1. Introduction

Conventional macromolecular crystallography at cryogenic temperatures (100 K) (cryo-MX) has so far been the silver bullet in determining structures of biomolecules at near-atomic resolution. This has been complemented with the advent of the ‘resolution revolution’ in cryo-electron microscopy (cryo-EM) (Kühlbrandt, 2014 ▸; Amunts *et al.*, 2014 ▸). However, structure determination under cryogenic conditions does not reveal biomolecular flexibility in native conformations (Fraser *et al.*, 2011 ▸; Fischer, 2021 ▸). Moreover, it does not permit the study of time-dependent structural changes coupled with enzymatic catalysis or protein dynamics (Keedy *et al.*, 2015 ▸; Weinert *et al.*, 2017 ▸; Doukov *et al.*, 2020 ▸; Fischer, 2021 ▸). Room-temperature crystallography (RT-MX) was initially proposed as a potential solution to this problem. In the early days, RT-MX was prone to high radiation damage and limited resolution due to the very low deposited dose allowed. Recent advances in serial crystallography at X-ray free-electron lasers (XFELs) and synchrotrons have rejuvenated RT-MX (Chapman *et al.*, 2011 ▸; Diederichs & Wang, 2017 ▸; Pearson & Mehrabi, 2020 ▸). Recent studies revealed that RT-MX in general has the potential to identify physiological conformations (Fraser *et al.*, 2011 ▸), allosteric networks (Keedy *et al.*, 2018 ▸), ligand-binding modes (Le Maire *et al.*, 2011 ▸; Gildea *et al.*, 2022 ▸) and fragment screening (Huang *et al.*, 2022 ▸). Moreover, RT-MX avoids the hassle of cryo-cooling crystals, which can be challenging for membrane proteins, viruses or large complexes while still yielding atomic resolution (Gavira *et al.*, 2020 ▸). Therefore, RT-MX has tremendous potential to improve structure-based drug discovery by facilitating a shortest path from protein to physiologically relevant structures. To this end, synchrotron facilities have already enabled RT data collection either through 96-well plates (Le Maire *et al.*, 2011 ▸; Axford *et al.*, 2012 ▸; Bingel-Erlenmeyer *et al.*, 2011 ▸; Doukov *et al.*, 2020 ▸), loops with sleeves (Russi *et al.*, 2017 ▸; Bowler *et al.*, 2015 ▸), *in meso in situ* COC chips for membrane proteins (Huang *et al.*, 2015 ▸, 2018 ▸), microfluidic chip (De Wijn *et al.*, 2021 ▸), serial synchrotron crystallography (Stellato *et al.*, 2014 ▸; Weinert *et al.*, 2017 ▸; Diederichs & Wang, 2017 ▸) or even dedicated beamlines for *in situ* experiments [*e.g.* VMXi at the Diamond Light Source (DLS) (Mikolajek *et al.*, 2023 ▸; Thompson *et al.*, 2024 ▸)]. RT-MX with *in situ* data collection is typically executed by measuring small wedges (10–60°) from single crystals in 96-well plates (Mikolajek *et al.*, 2023 ▸; Russi *et al.*, 2017 ▸). This requires visually inspecting each of the 96 drops, optical focusing with an on-axis camera and finally collecting diffraction data from selected positions (dependent on the drop position in the plate). Altogether, one 96-well plate can easily consume 6–8 h (*i.e.* a single-shift of allocated beam time). Thus, the traditional *in situ* data collection lacks high-throughput and efficiency, which in turn discourages users from their routine usage. This limits the application of *in situ* data collection to only diffraction-based screening of crystallization conditions of challenging projects. Here, we describe the first implementation of a plate gripper at a microfocus MX beamline: ID23-2 (Nanao *et al.*, 2022 ▸) at the European Synchrotron Radiation Facility (ESRF), enabling an easy setup, fast switching to and from standard cryo-setup, and rapid data collection by importing visual scores of drops executed in advance. We transformed *in situ* data collection into a serial crystallography approach by raster scanning each drop. Our so-called *in situ* serial crystallography (iSX) has been applied for the first time to determine a challenging structure in the *P*1 space group directly from a CrystalDirect (CD) plate (Cipriani *et al.*, 2012 ▸; Márquez & Cipriani, 2014 ▸) in order to establish the power of this approach. Thereby, iSX directly on 96-well plates can surmount the current ‘status quo’ of plate applications by providing a complete dataset as a by-product.

In this work, autotaxin from rat (r-ATX), which is a phospho­diesterase producing the lipid-signalling molecule lysophosphatidic acid (LPA), has been used as a challenging case study. ATX is the main producer of LPA, forming the ATX–LPA signalling axis with LPA acting as a multifunctional lipid mediator by engagement with six dedicated G-protein-coupled receptors (LPA_1–6_) (Eymery *et al.*, 2023 ▸). ATX regulates many pathophysiological processes [*e.g.* vascular development, neuropathy pain, fibrosis, rheumatoid arthritis, sclerosis and cancer (Moolenaar & Perrakis, 2011 ▸)]. The X-ray structure of r-ATX (MW 100 kDa) has already been determined through conventional cryo-MX (Hausmann *et al.*, 2011 ▸; Eymery *et al.*, 2023 ▸, 2024 ▸), giving a good control over crystallization, with minimal optimization to obtain sitting drops containing micro-crystals. r-ATX was expressed using a stable HEK293T cell line and 800 ml of cell culture yielded purified protein with a final concentration of ∼4 mg ml^−1^ (Eymery *et al.*, 2023 ▸), which is inadequate for batch crystallization. Thus, r-ATX serves as a relevant real-life case, justifying the viability of crystallization plates as the most natural sample delivery approach for serial crystallography experiments. However, application of iSX on micro-crystals facilitated the determination of the first RT structure of r-ATX at 3.0 Å in the *P*1 space group, with an effective data collection of <1.5 h.

## 2. Experimental setup at the ID23-2 beamline

**Figure 1:**
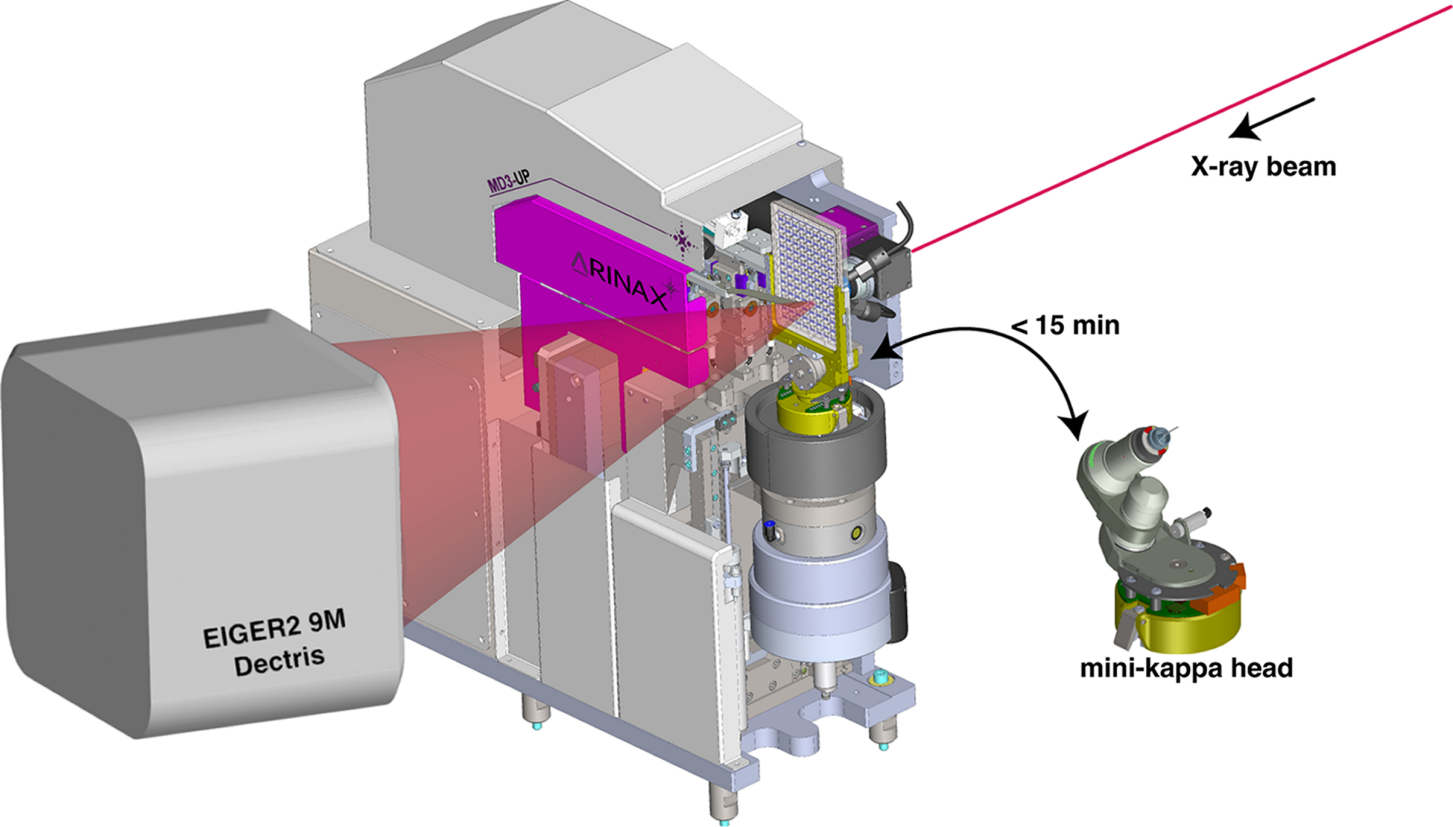
Schematic of the *in situ* experimental setup on ID23-2 with the PM installed on MD3-Up and a loaded CD plate. It also illustrates the fast switching from the conventional mini-kappa goniometer head to *in situ* setup (diffractometer drawing provided by Arinax, Moirans, France).

ID23-2 is a dedicated microfocus MX beamline that was completely rebuilt (Nanao *et al.*, 2022 ▸) to exploit the benefits of the extremely brilliant source (EBS) upgrade at the ESRF (Raimondi *et al.*, 2023 ▸). The beamline provides a fixed energy (14.2 keV) with variable micro-focusing from 2 to 10 µm^2^ beam size at a photon flux of 1.3 × 10^13^ photons s^−1^ at 200 mA ring-current and is equipped with a high-precision MD3-Up diffractometer (Arinax, Moirans, France) and EIGER2 9M detector (Casanas *et al.*, 2016 ▸). The experimental setup for *in situ* data collection uses high-precision features of the standard MD3-Up goniometer, complemented by an additional horizontal axis for aligning the crystallization plate on the omega axis of rotation. An attractive feature of the MD diffractometers is exchangeable goniometer heads. For cryo-MX, we typically use a mini-kappa 3 (MK3; Brockhauser *et al.*, 2013 ▸) goniometer head (Fig. 1 ▸), which can be easily switched for a dedicated plate holder, so-called plate manipulator (PM) for mounting SBS compatible 96-well plates in <15 min (Fig. 1 ▸). Both goniometer heads – MK3 and PM – utilize the same underlying translational and rotational stage to centre the crystal with the on-axis camera. In brief, given that the rest of the MD3-Up diffractometer functionality remains the same, the switching of portable goniometer heads allows for a rapid change from the cryo-MX to the *in situ* setup.

## 3. Integration of the plate-holder at the ID23-2 beamline

### 3.1. Plate manipulator for the micro-diffractometer

**Figure 2:**
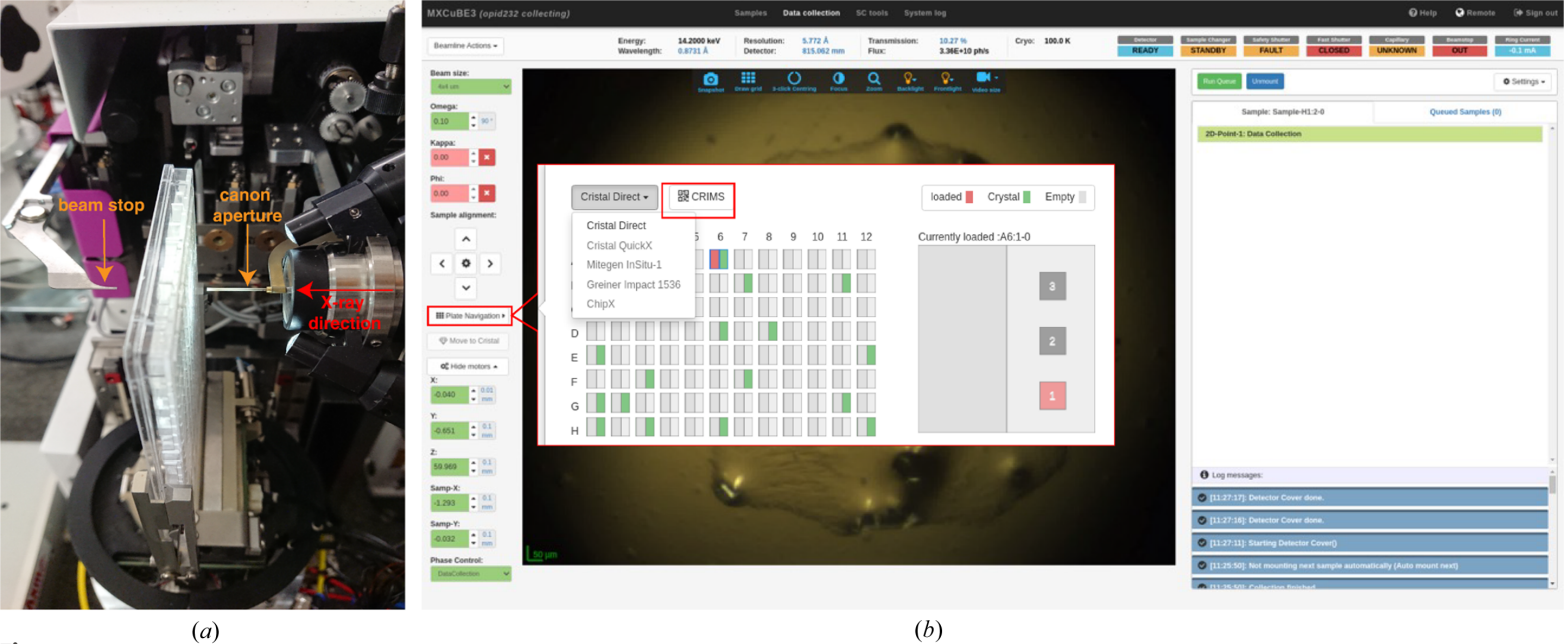
*In situ* data collection setup with a CD plate shown as mounted on ID23-2. (*a*) CD plate placed into the PM on an MD3-Up. (*b*) *MXCuBE-Web* interface to control *in situ* data collection from plates. A plate-navigator button is highlighted in red to emphasize the ease of going through different drops and the possibility to synchronize with the *CRIMS* database for the import of visual inspection scores (red inset). Additionally, a drop-down menu enlists different SB 96-well plates supported by the PM goniometer-head.

The PM is a hardware device with a fork-shaped design [Fig. 2 ▸(*a*)] compatible with the following SBS plates: *in situ*-1, Mitegen; Crystal-Direct, Mitegen; and CrystalQuick-X, Greiner Bio-One [Fig. 2 ▸(*b*)]. The PM horizontal axis for navigating on the plate relies on two different types of motion. The long-range motion is executed by a cable transmission driven by a stepper motor, while the short-range motion is performed by the centring-table stage of the micro-diffractometer. The centring-table is highly precise with <±2 µm within a 10 mm range and the effective resolution for crystal alignment is 300 nm. The vertical motion is performed by the MD3-Up high-precision vertical translation axis. The rotation capability has not been used in our protocol but is available through the omega rotation axis. The accessible omega range is up to 70° depending on the well location in the plate and the beam stop distance. Data collection can be performed over the whole area of the SBS plate regardless of the well position. It also allows the use of different data-collection procedures, using mesh, single or multi-point as well as pure raster scanning with a large range of oscillation. Similar to the *in situ* implementation on ID30B (McCarthy *et al.*, 2018 ▸), in order to exchange from the MK3 to the PM setup, the cryostream is removed and disabled, while the beam-defining aperture and capillary are replaced with a 20 mm-long and 100 µm-diameter canon aperture (a combined aperture and beam-cleaning capillary) [Fig. 2 ▸(*a*)]. Both goniometer heads at ID23-2 benefit from the latest version of the ‘quick lock’ mechanism to facilitate a rapid and reproducible positioning on the omega axis. A number of interlocks specific to the PM head are auto-configured to prevent potential collisions with diffractometer organs (*e.g.* backlight and beam stop). It takes ∼15 min to exchange goniometer heads and configure the MD3-Up for *in situ* data collection. The crystallization plates are manually mounted. However, remote experiments can be carried out on request. Executing a MeshScan over a 1 × 1 mm area requires ∼10 min.

### 3.2. Experimental workflow control software

#### 3.2.1. MXCuBE-Web

*MXCuBE-Web* is the latest generation of the data acquisition software *MXCuBE* [*Macromolecular Xtallography Customized Beamline Environment* (Oscarsson *et al.*, 2019 ▸)], a cross-facilities collaborative project in Europe. To facilitate *in situ* data collection, *MXCuBE-Web* has been enriched with new functionalities. Our implementation (described in the supporting information) considers the PM as a sample changer itself; each well is designated as a container and each drop represents a sample. The visual scores on the drops inside a 96-well plate, executed by users in their home laboratories, can easily be imported via an .XML file to *MXCuBE-Web*. Thus, users can directly navigate to the drops of interest with the corresponding crystals and subsequently collect diffraction data (Fig. 2 ▸) on a single or multiple crystals using different collection strategies available through *MXCuBE-Web*. In this work, we have simplified visual score import by exploiting the unique plate number attributed by the High-Throughput Crystallization (HTX) platform at the European Molecular Biology Laboratory (EMBL) Grenoble. This allows direct communication with the *Crystallization Information Management System* (*CRIMS*) to retrieve the drop indicated to contain crystals of interest. *CRIMS* is a web-based laboratory information system that provides automated communication between crystallization and synchrotron data collection facilities, enabling uninterrupted information flow over the whole sample life cycle from pure protein to diffraction data (Healey *et al.*, 2021 ▸).

#### 3.2.2. Micro-diffractometer software

The MD3-Up control software runs on a Windows PC and is based on the *JLib* library (EMBLEM Technology Transfer GmbH, Heidelberg, Germany; https://software.emblem.de). MD3-Up can be controlled using a graphical user interface (GUI) or remotely by a socket server using the *Exporter* protocol of *JLib*. The on-axis prosilica video camera of MD3-Up is read using the *LIMA* generic library for high-throughput image acquisition (Homs *et al.*, 2011 ▸). Within the MD3-Up control software a dedicated menu allows the user to navigate on the entire plate. Each type of plate benefits from its own layout for simple navigation.

## 4. Materials and methods

### 4.1. Micro-crystallization of rat autotaxin

Crystallization was performed using the HTX platform at EMBL (Grenoble) in CD plates. Crystallization drops were set up on an ultra-thin (<25 µm) COC-based film (TOPAS advanced polymers, USA) and the plate was sealed with ultra-thin polyolefin film (HJ-Bioanalytik GmbH). Thus, CD plates ensure minimal X-ray background (Fig. S1 of the supporting information) during *in situ* data collection, unlike ‘classic’ plastic materials found in most crystallization plates. Crystals were obtained using an adapted protocol of the conditions previously described for r-ATX-beta (Eymery *et al.*, 2023 ▸). The r-ATX-beta protein solution was concentrated to 3–4 mg ml^−1^ in Tris 50 m*M*, NaCl 150 m*M* at pH 8. The best diffracting microcrystals were obtained in 20–32% PEG3350, 0.1–0.4 M NH_4_I, 0.1–0.4 M NaSCN using the hanging-drop method. Typically, higher concentrations of PEG3350 resulted in smaller crystals with higher density per drop. In each CD plate, 96 wells were filled with precipitant and three drops per condition were set up at different protein:precipitant ratios (1:1, 1:2 and 1:3). Promising drops (Fig. S2) with high microcrystal density were ranked for data collection after automated visible and UV light imaging, and visual inspection scores were registered in the *CRIMS* database.

### 4.2. *In situ* serial crystallography data collection from a CrystalDirect plate

At ID23-2, data collection is conducted via *MXCuBE-Web* (Oscarsson *et al.*, 2019 ▸) and results/output of data collection and automated processing are displayed in the data-management system *EXI-ISPyB* (Delagenière *et al.*, 2011 ▸). *MXCuBE-Web* has been adapted to support *in situ* (RT) data collection from plates. *MXCuBE-Web* in ‘plate mode’ enables users to import visual inspection scores for each CD plate, previously conducted at the EMBL-Grenoble HTX facility, by synchronizing with the *CRIMS* database (Cornaciu *et al.*, 2021 ▸; Healey *et al.*, 2021 ▸) [Fig. 2 ▸(*b*)]. We collected ∼5.7 million diffraction frames from 61 drops, across 2 CD plates using *MeshScan* (Zander *et al.*, 2015 ▸), a modular automated ESRF workflow system (*EWOKS*) routine available through *MXCuBE-Web*. An example diffraction frame from an r-ATX microcrystal together with a quantitative analysis on the background contribution by the crystallization plate are provided in Fig. S1. Each drop was raster scanned with 4 × 4 µm beam size X-rays at 14.2 keV photon energy, a flux of 7 × 10^11^ photons s^−1^ (*i.e.* 10% of full flux) and 6 ms exposure per frame without any rotation. This *in situ* serial data collection strategy yielded ∼100 000 (average) still diffraction frames per drop in ∼10 min. As the drop is continuously exposed to X-rays during the raster-scan, a deposited dose is accumulated across the entire drop. This, in turn, may form radicals inside the crystallization buffer, which cannot be taken into account by *RADDOSE-3D* (Zeldin *et al.*, 2013 ▸). Therefore, we empirically devised a data collection strategy that delivers a diffraction weighted dose of ∼35 kGy per diffraction frame, as calculated using *RADDOSE-3D*, which does not lead to site-specific damage.

### 4.3. Cryogenic serial data collection on autotaxin

Diffraction data were collected at ID23-2 at the ESRF, Grenoble, France (Nanao *et al.*, 2022 ▸). Measurements from r-ATX microcrystals were carried out under cryogenic conditions (100 K) with a 4 × 4 µm microfocus beam at 14.2 keV (0.8731 Å wavelength). Entire crystallization drops were harvested and raster scanned with X-ray exposure times of 6 ms per image to identify positions corresponding to well diffracting crystals. In a second step, 10° wedges consisting of 100 frames with 0.1° oscillation were collected at a photon flux of 1.4 × 10^12^ photons s^−1^ with a 20 ms per frame exposure time from each of the identified positions at a speed of 5° s^−1^ using the *Mesh&Collect* automated multi-crystal data collection workflow (*EWOKS*) (Zander *et al.*, 2015 ▸). This strategy ensured the diffraction-weighted dose to be 3 MGy per sweep, as calculated by *RADDOSE-3D* (Zeldin *et al.*, 2013 ▸). We examined qualitatively if there is a loss of intensity at high resolution to determine the permissible dose in each sweep. If dose allowed, 2–3 sweeps (*i.e.* ∼6–9 MGy) of 10° wedges were collected from each crystal identified. Each 10° wedge takes typically 2 s. This ensured redundant data collection from each microcrystal. During data processing, sweeps that suffered from high deposited dose were not successfully indexed/integrated and hence were excluded from the scaling and merging steps (see Section 5).

## 5. Data processing, structure determination and refinement

**Figure 3:**
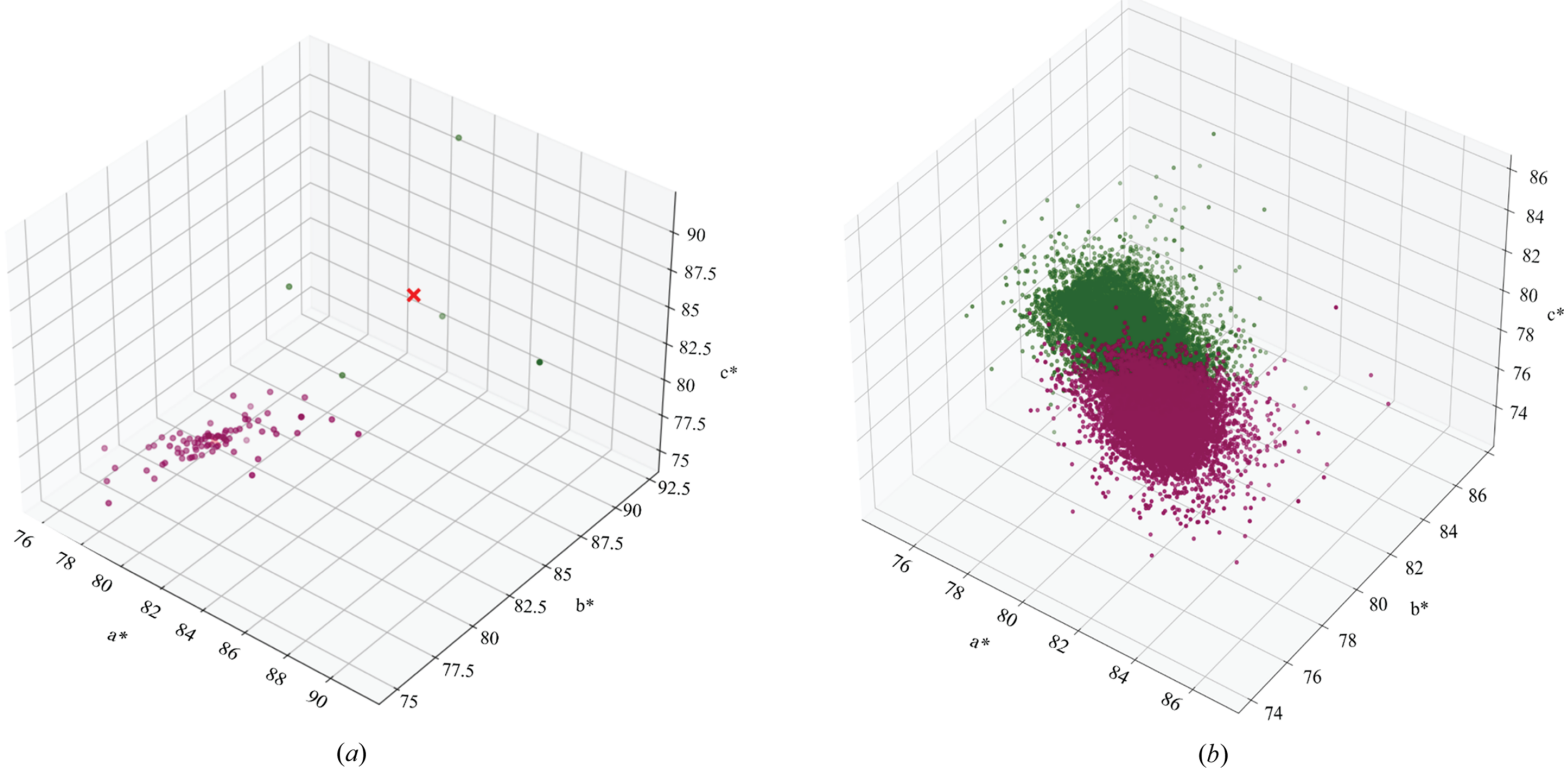
Final distribution of crystal unit-cell dimensions from the cryo-SSX and iSSX datasets, shown in panels (*a*) and (*b*), respectively. Each dot represents a crystal lattice. For visualization, unit cells were orthogonalized in reciprocal space. In 3D Cartesian coordinates, each dot is associated with a*, b*, c* vector cell along the *x*, *y*, *z* axes, respectively (with units in Å^−1^). a*, b*, c* have been calculated using the cosine law formula (**ab**^2^)^1/2^, (**bc**^2^)^1/2^, (**ca**^2^)^1/2^. The two populations are shown in green and purple using the *k-means* clustering method.

(1) Cryo-SSX data processing. Initial diffraction patterns, collected under cryogenic condition at ID23-2, were processed using *XDS* (Kabsch, 2010 ▸), followed by real time automated selection and merging of statistically equivalent datasets with *XSCALE* using the *sxdm* tool (Basu *et al.*, 2019 ▸). Datasets were selected using a combination of multiple criteria, including an ISa cutoff of 3.0, unit cell and pair-CC based hierarchical clustering. We provided a *k-means* analysis [Fig. 3 ▸(*a*)] on unit-cell axes in reciprocal space (i.e.a*, b*, c*) to show a tight-cell distribution for the selected datasets. This allowed us to quickly identify 91 statistically equivalent mini-datasets (*i.e.* 10° wedges), which were then scaled and merged using *XSCALE*. Thus, a reflection file (MTZ format) was produced for further structural analysis. All the structures were solved by molecular replacement (MR) using *PHASER* (McCoy, 2007 ▸) with the PDB entry 4zga (Stein *et al.*, 2015 ▸) as the search model. The resolution cutoff was determined to be 2.2 Å using a combined metric of CC_1/2_ ≥ 0.30 and *I*/σ(*I*) ≥ 1.0 (Karplus & Diederichs, 2012 ▸). Structure refinement was carried out iteratively with *phenix.refine* (Afonine *et al.*, 2012 ▸) and *Coot* (Emsley *et al.*, 2010 ▸). *MolProbity* (Williams *et al.*, 2018 ▸) was used to assess the quality of the structure and the data collection and the refinement statistics are summarized in Table 1 ▸.

(2) iSX data processing. The still diffraction frames [Fig. S1(*a*)] collected at RT were processed using *crystFEL* (version 0.10.1; White *et al.*, 2012 ▸) distributed within *SBGrid* (Morin *et al.*, 2013 ▸). Peak-finding was done using the *peakfinder8* algorithm (Barty *et al.*, 2014 ▸). Indexing was performed with *indexamajig* combined with *xgandalf* (Gevorkov *et al.*, 2019 ▸) and *mosflm* (Battye *et al.*, 2011 ▸). Data were merged at the resolution cutoff of 3.0 Å using *partialator* with the *unity* model (*CrystFEL*). The statistics reported (Table 1 ▸) have been calculated using *check_hkl* and *compare_hkl* (*CrystFEL*). *TRUNCATE* (French & Wilson, 1978 ▸) from *ccp4i* (Agirre *et al.*, 2023 ▸) was used to convert intensities (*I*) to structure factors (SFs). The structure was determined by molecular replacement using *PHASER* (Read, 2001 ▸) from *phenix* (Adams *et al.*, 2010 ▸) with the PDB entry 4zga (Stein *et al.*, 2015 ▸) as a reference model, homologous at 92.2% in sequence calculated with *SIM* (Duret *et al.*, 1996 ▸) from the *expasy.org* web server. The reference model was prepared using *PDBSET* (*ccp4i*). Refinement was carried out using *phenix.refine* (Afonine *et al.*, 2012 ▸) for reciprocal space and *Coot* (Emsley *et al.*, 2010 ▸) for real space, yielding *R*_free_/*R*_work_ values of 0.28/0.22, respectively.

### 5.1. Space group and unit-cell determination

As r-ATX was crystallized in a triclinic lattice, unit-cell determination on still diffraction frames obtained from millions of different microcrystals was challenging. Primarily, indexing on each drop containing a reasonably high number of crystal hits was executed with the *indexamajig* program within *CrystFEL* using the *mosflm* and *xgandalf* algorithms. The diffraction frames, indexed in a triclinic lattice, were clustered in multiple unit-cell parameters. Using these initial results, we were able to find three ‘crystal populations’, out of which, two populations appeared more isomorphous than the third one. Thus, we determined two sets of cell parameters (cell-I) *a*, *b*, *c* (Å): 54.16, 62.66, 65.55, and α, β, γ (°): 77.65, 81.24, 93.80; and (cell-II) *a*, *b*, *c* (Å): 53.5, 61.19, 65.01, and α, β, γ (°): 102.56, 99.12, 93.67. We used these cell parameters to repeat the indexing step. The indexing performed with cell-II parameters had a better success rate compared with indexing executed with cell-I. We therefore focused on the results of indexing obtained with cell-II parameters. Subsequently, we performed indexing with empirically determined unit-cell tolerance parameters of 12% on the a*, b* and c* axes and 5% on angles. This strategy gave us the best results with 29 553 indexed frames from a total of 5 557 065 hits detected over 13 790 703 diffraction frames collected. The final indexing results appeared to be slightly non-isomorphic, which was investigated using the *k-means* analysis (Fig. 3 ▸) to determine the degree of heterogeneity. Fig. 3 ▸(*b*) quantified that the two suspected populations of crystal cells were actually very close to each other with an Euclidean vector distance of 3.28 Å, which corresponds to a negligible difference of 0.09% in the unit-cell volume. To complete the data analysis, we further visualized Bragg reflections in 3D using the *phenix data 3D viewer* tool and observed no anisotropicity due to preferred orientation, which would normally be expected from r-ATX crystal morphology (Fig. S3).

### 5.2. Merging and resolution cutoff

Indexed frames were merged using *partialator* in *CrystFEL*. The resolution of the merged dataset was truncated at 3.0 Å considering the behaviour of *R*_split_, CC*, CC and 〈*I*/σ(*I*)〉. All the data processed for the *a posteriori* analysis has been truncated at 3.0 Å resolution to be directly comparable with our reference dataset. Our ‘conservative’ resolution cutoff was decided based on the heterogeneity due to different crystals with variable sizes and diffraction quality. The variability within the sample has an impact on the proper estimation of the error modelling of the intensity measurements. We took this information into account and decided to cut the data at a relatively high 〈*I*/σ(*I*)〉 value (Wlodawer *et al.*, 2008 ▸; Evans & Murshudov, 2013 ▸).

### 5.3. Optimizing future experiments by *a posteriori* analysis

**Figure 4:**
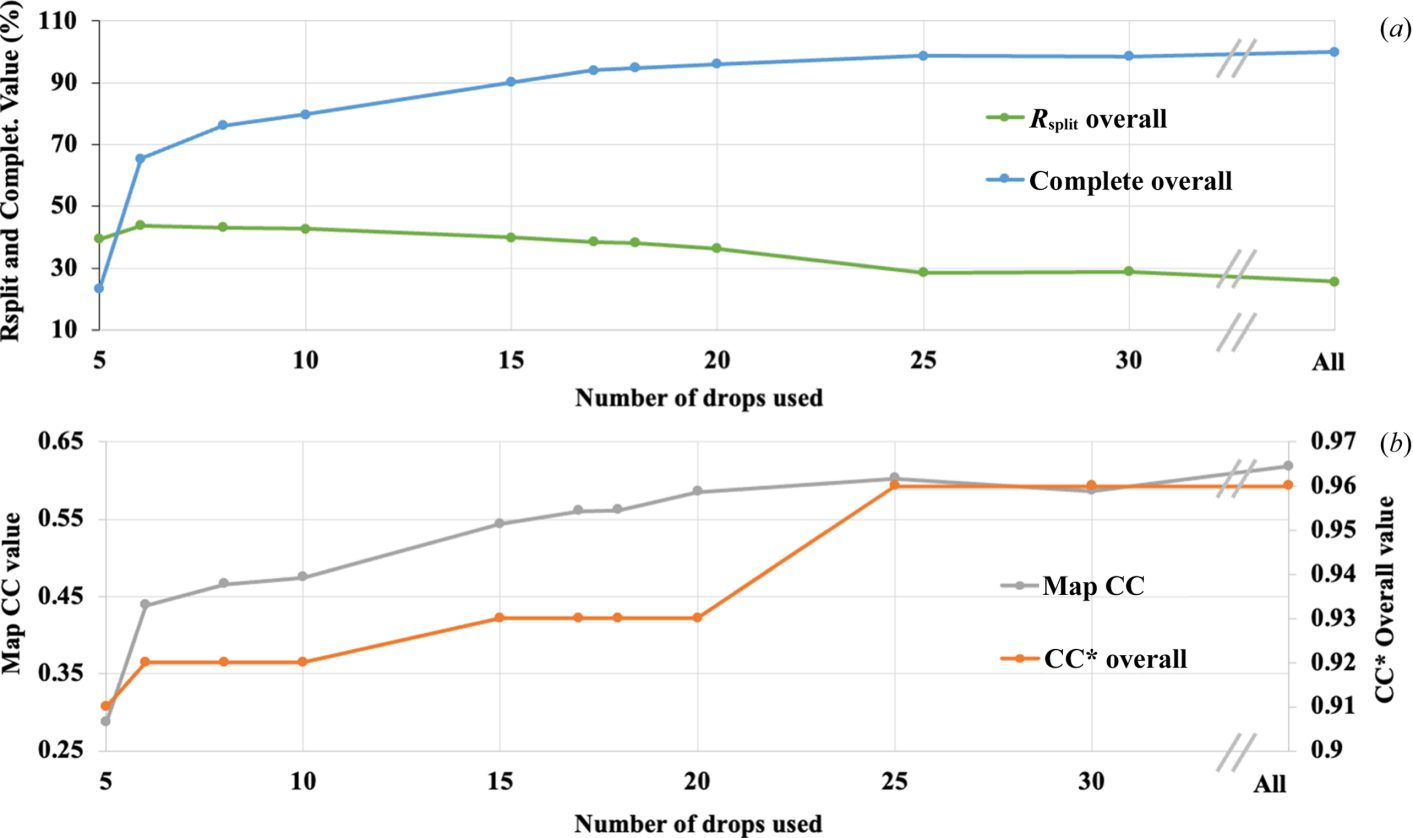
Merging statistics of iSX datasets plotted against the number of drops for each dataset, from 5 to 30 drops, and comparison of each dataset used for refinement. Panel (*a*): in green: *R*_split_ overall (%); in cyan: completeness (%). Panel (*b*): in orange CC* overall (right axis); in grey: map correlation (left axis) between the first 2*mF*_o_ − *DF*_c_ electron density map obtained after molecular replacement and final refined ‘All’, implying 61 drops, 2*mF*_o_ − *DF*_c_ electron density map.

**Figure 5:**
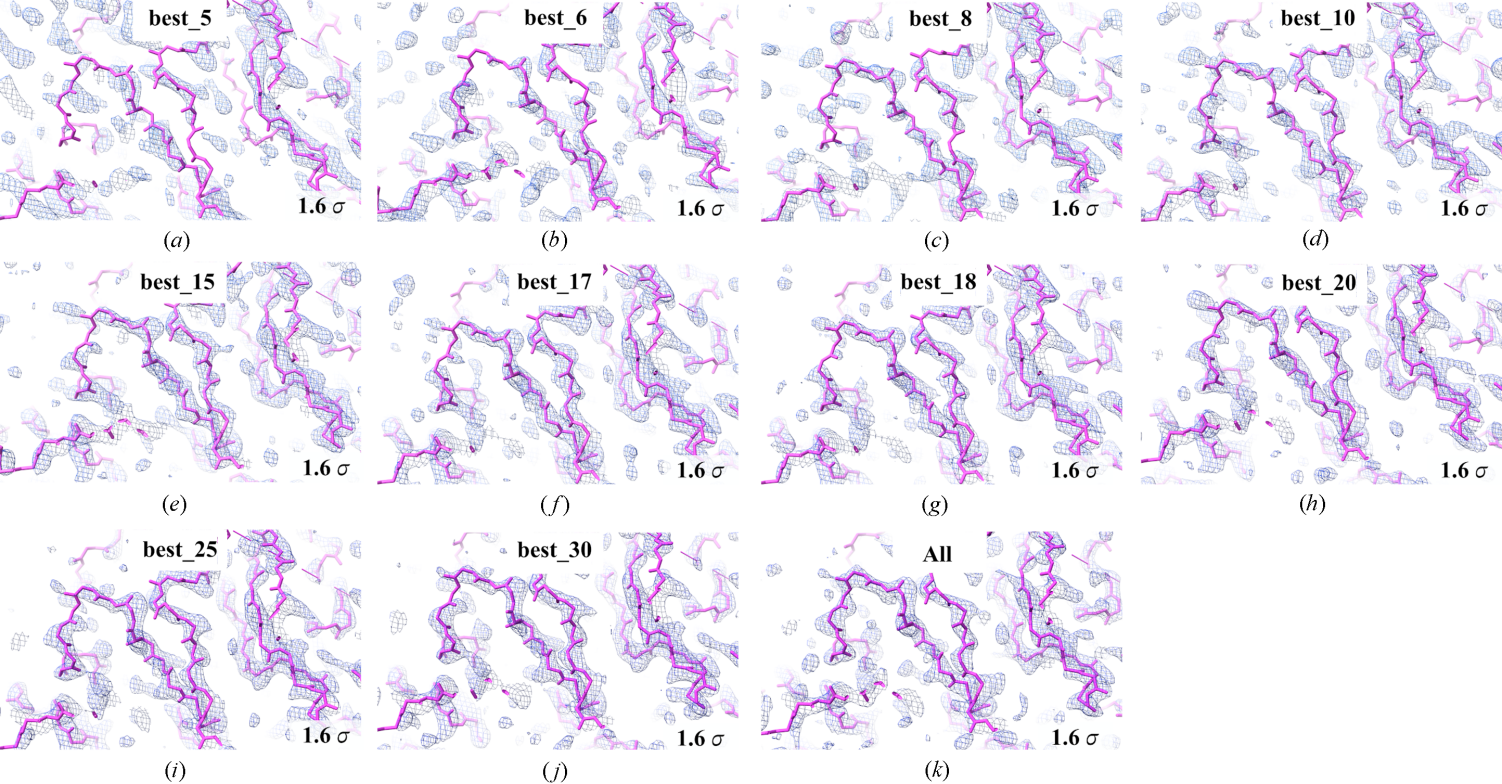
iSX structure of r-ATX solved in the space group *P*1. Comparison of the first 2*F*_o_ − *F*_c_ electron density map at the 1.6σ level from the molecular replacement result produced by *phaser* and displayed as a blue mesh overlaid onto the r-ATX Cα model (pink sticks). (*a*) best_5, (*b*) best_6, (*c*) best_8, (*d*) best_10, (*e*) best_15, (*f*) best_17, (*g*) best_18, (*h*) best_20, (*i*) best_25, (*j*) best_30 and (*k*) all-data: 61. Qualitatively the electron density is easily interpretable from (*d*) best_10 onwards.

An *a posteriori* analysis of our data was executed to determine an optimal beam time session for a large triclinic structure. To this end, we ranked the drops in descending order based on the number of indexed frames, followed by merging. Thus, we estimated the minimum number of drops required to obtain a complete dataset for r-ATX in the triclinic lattice. We prepared 10 datasets by merging indexed frames from the ‘best’ 5, 6, 8, 10, 15, 17, 18, 20, 25 and 30 drops. Using this method, we determined that after iSX data collection on 10 drops we had a usable dataset, and after 17 drops the CC* began to converge close to the value obtained from all-data, comprising 61 drops (Fig. 4 ▸). Molecular replacement (MR) was performed for all sub-datasets using the same model as for the original phasing. Subsequently, the MR solutions were compared to visually inspect the electron density interpretability for each dataset (Fig. 5 ▸). From the best_10 dataset onwards, manual model building in real space was clearly possible. This corresponds to the effective data collection time of 1.5 h across 10 drops.

### 5.4. Structure comparison between iSX and cryo-SSX

**Figure 6:**
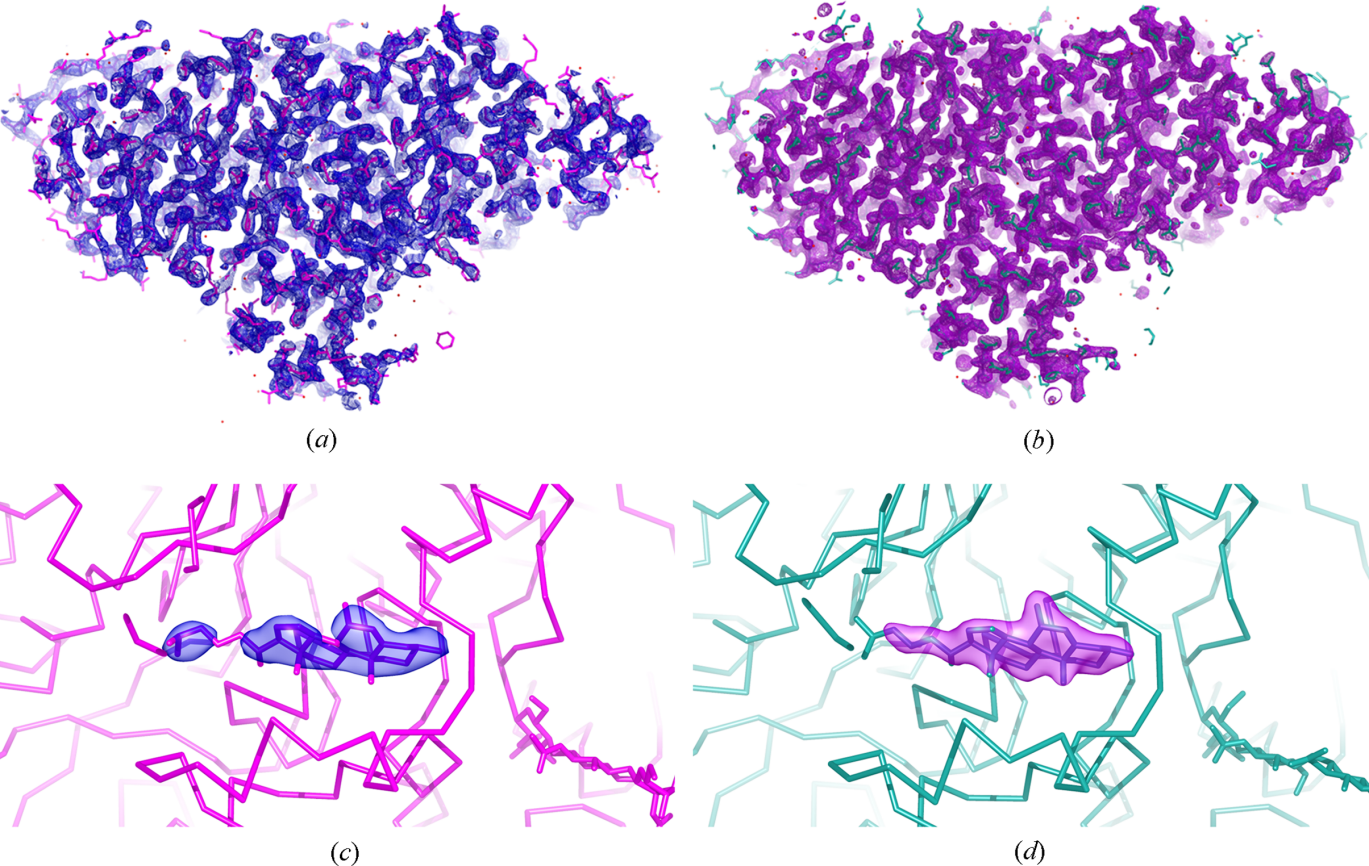
Serial crystallography structure of r-ATX in the space group *P*1. (*a*) and (*c*) display the *in situ* determined structure, (*b*) and (*d*) display the structure determined from serial cryo data collection. (*a*) Overall 2*F*_o_ − *F*_c_ electron density map at the 1.2σ level in blue overlaid onto the r-ATX model in the magenta sticks representation. (*c*) 2*F*_o_ − *F*_c_ electron density map in blue for the oxysterol moiety bound in the allosteric tunnel of the r-ATX model shown in magenta. (*b*) Overall 2*F*_o_ − *F*_c_ electron density map at the 1.2σ level in purple overlaid onto the r-ATX model in ocean green sticks representation. (*d*) 2*F*_o_ − *F*_c_ electron density map in purple for the oxysterol moiety bound in the allosteric tunnel of r-ATX model shown in ocean green.

We compared the iSX and cryo-SSX structures of r-ATX by superimposition and RMSD calculation using *pymol* (DeLano, 2002 ▸) (Figs. 6 ▸ and S4). Upon structural comparison between iSX and cryo-SSX models of r-ATX, we did not observe any differences at the active site. However, both – iSX and cryo-SSX – structures reveal an oxysterol moiety bound in the allosteric tunnel (Fig. 6 ▸). We compared the relative *B*-factor change (∂*B*_relative_) between the iSX and cryo-SSX structures of r-ATX. For each model, the *B*-factors were averaged over all residues followed by subtraction of the *B*-factor of the cryo-SSX model from the *in situ* model for each residue. We applied a fixed positive offset over all the values to have the lowest value at zero. We then used *PyMOL* to represent the *B*-factor difference, combining putty and rainbow colour representation (Fig. S4) (DeLano, 2002 ▸). We calculated the overall RMSD between the two models, resulting in a value of 1.34 Å (Fig. S4). We observed the largest RMSD value for small domains [Fig. S4(*a*)] outside the protein core in regions that are located along the solvent channel, considering the crystal packing [Fig. S4(*b*)]. The larger volume in such a location allows a wider distribution of possible loop conformations. The *B*-factor difference between *in situ* and cryo-structures follows the same trend as the RMSD. The higher *B*-factor differences are colocalizing in more flexible domains at the protein surface in the crystal contact regions.

## 6. Discussion

This work combines the advantages of conventional high-throughput crystallization in plates with the rapidity of serial crystallography and demonstrates the strength and efficacy of iSX, even for difficult cases. Our iSX approach relies on easy and rapid raster scanning at any modern microfocus beamline, such as ID23-2, equipped with a state-of-the-art micro-diffractometer, MD3-Up and fast data acquisition software. The iSX strategy may be envisaged to help users of a novel target without any prior information about the diffraction quality while potentially acquiring a complete dataset. An alternative approach that could have been applied is the ‘*Mesh&Collect*’ protocol, which has the advantages of including small rotations, but has the disadvantages of longer data collection times and general impracticality at RT. However, such a strategy can be successful at RT only at very low doses for both determining the diffractive map and for mini-rotations. One could reduce the dose significantly, but depending on how the dose budget is spent this can be extremely difficult to process with conventional MX software. Interestingly, on visual inspection of Bragg reflections from the iSX dataset in 3D reciprocal volume, the data appeared to be complete up to 3 Å. Similar to the cryo-SSX dataset, preferred orientation was not observed [Figs. S3(*a*)–S3(*b*)] in the iSX dataset, even though microcrystals were plate shaped. Such crystal morphology would normally be expected to have an orientation bias. Importantly, most of the challenging large protein complexes (*e.g.* r-ATX) are expressed in mammalian cells, typically yielding a concentration of a few milligrams per millilitre of purified protein. Moreover, scaling up the protein production for higher eukaryotic expression systems can be a very expensive and time-consuming process. Microcrystallization for such cases through batch methods is not suitable; this seems to be a bottleneck in expanding serial crystallography (Beale *et al.*, 2019 ▸) on the many challenging medically relevant targets (*e.g.* membrane proteins). Thus, a 96-well crystallization plate might be able to serve as a more efficient fixed-target sample delivery tool for serial crystallography experiments. Our iSX approach is easy, rapid and potentially reduces preferred orientation issues because crystals are not deposited on solid supports. Our work also shows that iSX can tackle even the most challenging target, *i.e* small crystals that do not diffract to high resolution.

We collected still images by raster scanning an X-ray microbeam while continuously moving the plate without rotation. This implied data processing with serial crystallography software (*e.g.CrystFEL*). We deliberately chose a set of parameters for the hit finding that were tolerant, resulting in a large number of images considered as hits (more false positive), many of which could not be indexed in the subsequent steps. As we did not benefit from any rotation information, the indexing has been quite challenging for the triclinic lattice. However, we managed to iteratively refine our cell parameters from an initial heterogeneous distribution of cell parameters. Subsequently, 29 550 indexed diffraction frames were merged using the *partialator* program within *CrystFEL*. We determined the *in situ* r-ATX structure at 3 Å in the *P*1 space group using molecular replacement, followed by refinement in *Phenix*. The iSX dataset is limited to a lower resolution compared with the cryo-SSX one mainly due to a low deposited dose at RT.

In addition, we determined the r-ATX structure using cryo-SSX by collecting 10° wedges on many micro-crystals via the *Mesh&Collect* strategy. This served as a reference structure that was used for comparison with the iSX model. Superimposition of the two models showed no large overall structural differences, except variations that occurred in crystal-contact regions [Fig. S4(*b*)]. This observation supports other studies, revealing that RT structures are more flexible and dynamic, potentially representing a more physiological state (Fraser *et al.*, 2011 ▸). Furthermore, we compared the *B*-factor variation per residue between the two models and showed that the variations are again in the region where the crystal packing is less constrained [Fig. S4(*c*)]. As a result, this demonstrates that structures determined via iSX are at least as informative as those resulting from cryogenic temperature and may provide structural insights into flexible domains or regions.

To demonstrate the efficiency of our method, an *a posteriori* data analysis was performed aimed at determining the optimal data collection strategy. Thus, we deduced that we had an acceptable dataset after merging indexed frames from 10 drops by evaluating the overall completeness (Fig. 4 ▸ and Table 1 ▸). Fig. 5 ▸ further supports the results visually through the electron density maps. The statistics and electron densities were improved by adding more indexed images and we decided to use ‘drops’ as data collection units instead of image numbers to discuss the efficiency. This is consistent with the fact that, in our experimental setup, the fast data collection is performed drop by drop. We expect users to start with the most promising drops in terms of crystal density/quality. This justifies our methodology to accumulate diffraction frames starting from the promising drops which contained good crystal density. Here, we focus on the effective data collection time required to obtain a minimal dataset for the structural determination of a large biomolecule of ∼100 kDa in triclinic symmetry. Thus, it can be deduced that such a large triclinic structure could be determined from a minimum of 10 crystallization drops in less than 1.5 h (∼6% of a single beam time shift) of effective data collection. In the case of iSX data collection of r-ATX at ID23-2, it is noteworthy that the sampling of the drop using raster scans is driven by the beam size, implying a very large number of diffraction frames with a microfocus beam. This had a side effect on the number of ‘empty’ images, which may yield an ‘artificially’ low indexing rate for such a large dataset (Table 1 ▸).

To the best of our knowledge, this work was the first success in an attempt to determine the molecular structure of a large protein in the lowest possible symmetry using an iSX approach. In addition, our iSX approach requires a very small amount of sample (3 µg of protein, Table 1 ▸) and an efficient data collection time compared with other existing SSX-based sample delivery approaches (Weinert *et al.*, 2017 ▸; Moreno-Chicano *et al.*, 2019 ▸), which can easily take several hours for such a large protein in a low-symmetry space group. The ease and rapidity of our iSX strategy, we believe, will make *in situ* data collection under ambient conditions high-throughput and universally applicable to other MX beamlines. Importantly, iSX promotes crystallization plates as an alternative, efficient and the most natural approach to delivering samples for serial crystallography experiments at synchrotrons.

## Supplementary Material

PDB reference: SSX structure of autotaxin under cryogenic conditions, 9ent

PDB reference: SSX structure of autotaxin at room temperature, 9eu5

Supporting information and figures. DOI: 10.1107/S2052252524005785/lz5073sup1.pdf

## Figures and Tables

**Table 1:** Data collection and refinement statistics for r-ATX Values in parentheses represent the highest-resolution shell.

	r-ATX – iSX	r-ATX – cryo-SSX	r-ATX – iSX (10 drops)
Data availability	https://doi.org/10.15151/ESRF-DC-1565254585	https://doi.org/10.15151/ESRF-DC-1562957625	
PDB entry	9eu5	9ent	

Data statistics			
Space group	*P*1	*P*1	*P*1
*a*, *b*, *c* (Å)	54.51, 61.19, 65.001	53.51, 61.19, 65.01	54.51, 61.19, 65.001
α, β, γ (°)	102.568, 99.128, 93.67	102.568, 99.128, 93.67	102.568, 99.128, 93.67
Effective sample consumption (µg)	18	3.3	3
No. of diffraction frames collected	13790703	934 × 100	982274
No. of crystal hits	5557065	–	122873
No. of indexed frames merged	29553	91 × 100	5132
Effective data collection time (h)	9	<1	1.5
Resolution range (Å)	62.448–3.0 (3.14–3.04)	37.51–2.2 (2.27–2.2)	62.448–3.0 (3.14–3.04)
Total No. of reflections	2139506 (116235)	270477 (19435)	309655 (16378)
No. of unique reflections	32245 (2669)	34797 (2597)	25737 (1946)
Completeness (%)	99.96 (99.89)	99.96 (99.89)	79.79 (72.83)
Multiplicity	66.35 (43.6)	7.77 (7.48)	12.01 (8.4)
〈*I*/σ(*I*)〉	4.31 (1.79)	4.45 (0.97)	2.64 (0.95)
CC_1/2_	0.87 (0.56)	0.952 (0.40)	0.74 (0.44)

Refinement statistics			
Resolution range (Å)	62.45–3.00	37.51–2.2	62.45–3.00
No. of unique reflections/test set	16,129/825	39724/1986	14768/738
*R*_free_/*R*_work_†	0.2809/0.2245	0.2357/0.1938	0.3393/0.2986
No. of atoms	6688	6968	6563
Protein	6354	6424	6344
Others	131	95	125
Water	203	449	94
*B* factors			
Wilson plot	59.34	38.32	73.34
Total	55.31	47.33	68.14
Protein	55.17	46.93	68.44
Water	44.91	49.7	31.88
Others	78.11	63.18	35.07
Root-mean-square deviation‡			
Bond lengths (Å)	0.003	0.003	0.014
Bond angles (°)	0.588	0.643	1.922
Ramachandran			
Favoured (%)	95.52	96.02	93.07
Outliers	0	0	9

† Crystallographic *R* factor, *R*_work_ = Σ ||*F*_o_| − |*F*_c_||/Σ|*F*_o_|, where *F*_o_ and *F*_c_ are the observed and calculated structure factors, respectively. *R*_free_ is *R*_work_ calculated for a randomly selected subset of 5% of the data for the r-ATX crystals, not used in the refinement procedure.

‡ Root-mean-square deviations from the standard values for bond lengths and angles.

## References

[bb1] Adams, P. D., Afonine, P. V., Bunkóczi, G., Chen, V. B., Davis, I. W., Echols, N., Headd, J. J., Hung, L.-W., Kapral, G. J., Grosse-Kunstleve, R. W., McCoy, A. J., Moriarty, N. W., Oeffner, R., Read, R. J., Richardson, D. C., Richardson, J. S., Terwilliger, T. C. & Zwart, P. H. (2010). *Acta Cryst.* D**66**, 213–221. 10.1107/S0907444909052925 PMC2815670 20124702

[bb2] Afonine, P. V., Grosse-Kunstleve, R. W., Echols, N., Headd, J. J., Moriarty, N. W., Mustyakimov, M., Terwilliger, T. C., Urzhumtsev, A., Zwart, P. H. & Adams, P. D. (2012). *Acta Cryst.* D**68**, 352–367. 10.1107/S0907444912001308 PMC3322595 22505256

[bb3] Agirre, J., Atanasova, M., Bagdonas, H., Ballard, C. B., Baslé, A., Beilsten-Edmands, J., Borges, R. J., Brown, D. G., Burgos-Mármol, J. J., Berrisford, J. M., Bond, P. S., Caballero, I., Catapano, L., Chojnowski, G., Cook, A. G., Cowtan, K. D., Croll, T. I., Debreczeni, J. É., Devenish, N. E., Dodson, E. J., Drevon, T. R., Emsley, P., Evans, G., Evans, P. R., Fando, M., Foadi, J., Fuentes-Montero, L., Garman, E. F., Gerstel, M., Gildea, R. J., Hatti, K., Hekkelman, M. L., Heuser, P., Hoh, S. W., Hough, M. A., Jenkins, H. T., Jiménez, E., Joosten, R. P., Keegan, R. M., Keep, N., Krissinel, E. B., Kolenko, P., Kovalevskiy, O., Lamzin, V. S., Lawson, D. M., Lebedev, A. A., Leslie, A. G. W., Lohkamp, B., Long, F., Malý, M., McCoy, A. J., McNicholas, S. J., Medina, A., Millán, C., Murray, J. W., Murshudov, G. N., Nicholls, R. A., Noble, M. E. M., Oeffner, R., Pannu, N. S., Parkhurst, J. M., Pearce, N., Pereira, J., Perrakis, A., Powell, H. R., Read, R. J., Rigden, D. J., Rochira, W., Sammito, M., Sánchez Rodríguez, F., Sheldrick, G. M., Shelley, K. L., Simkovic, F., Simpkin, A. J., Skubak, P., Sobolev, E., Steiner, R. A., Stevenson, K., Tews, I., Thomas, J. M. H., Thorn, A., Valls, J. T., Uski, V., Usón, I., Vagin, A., Velankar, S., Vollmar, M., Walden, H., Waterman, D., Wilson, K. S., Winn, M. D., Winter, G., Wojdyr, M. & Yamashita, K. (2023). *Acta Cryst.* D**79**, 449–461.

[bb4] Amunts, A., Brown, A., Bai, X., Llácer, J. L., Hussain, T., Emsley, P., Long, F., Murshudov, G., Scheres, S. H. W. & Ramakrishnan, V. (2014). *Science*, **343**, 1485–1489. 10.1126/science.1249410 PMC4046073 24675956

[bb5] Axford, D., Owen, R. L., Aishima, J., Foadi, J., Morgan, A. W., Robinson, J. I., Nettleship, J. E., Owens, R. J., Moraes, I., Fry, E. E., Grimes, J. M., Harlos, K., Kotecha, A., Ren, J., Sutton, G., Walter, T. S., Stuart, D. I. & Evans, G. (2012). *Acta Cryst.* D**68**, 592–600. 10.1107/S0907444912006749 PMC4791750 22525757

[bb6] Barty, A., Kirian, R. A., Maia, F. R. N. C., Hantke, M., Yoon, C. H., White, T. A. & Chapman, H. (2014). *J. Appl. Cryst.* **47**, 1118–1131. 10.1107/S1600576714007626 PMC4038800 24904246

[bb7] Basu, S., Kaminski, J. W., Panepucci, E., Huang, C.-Y., Warshamanage, R., Wang, M. & Wojdyla, J. A. (2019). *J. Synchrotron Rad.* **26**, 244–252. 10.1107/S1600577518016570 PMC6337882 30655492

[bb8] Battye, T. G. G., Kontogiannis, L., Johnson, O., Powell, H. R. & Leslie, A. G. W. (2011). *Acta Cryst.* D**67**, 271–281. 10.1107/S0907444910048675 PMC3069742 21460445

[bb9] Beale, J. H., Bolton, R., Marshall, S. A., Beale, E. V., Carr, S. B., Ebrahim, A., Moreno-Chicano, T., Hough, M. A., Worrall, J. A. R., Tews, I. & Owen, R. L. (2019). *J. Appl. Cryst.* **52**, 1385–1396. 10.1107/S1600576719013517 PMC6878878 31798361

[bb10] Bingel-Erlenmeyer, R., Olieric, V., Grimshaw, J. P. A., Gabadinho, J., Wang, X., Ebner, S. G., Isenegger, A., Schneider, R., Schneider, J., Glettig, W., Pradervand, C., Panepucci, E. H., Tomizaki, T., Wang, M. & Schulze-Briese, C. (2011). *Cryst. Growth Des.* **11**, 916–923.

[bb11] Bowler, M. W., Mueller, U., Weiss, M. S., Sanchez-Weatherby, J., Sorensen, T. L.-M., Thunnissen, M. M. G. M., Ursby, T., Gobbo, A., Russi, S., Bowler, M. G., Brockhauser, S., Svensson, O. & Cipriani, F. (2015). *Cryst. Growth Des.* **15**, 1043–1054.

[bb12] Brockhauser, S., Ravelli, R. B. G. & McCarthy, A. A. (2013). *Acta Cryst.* D**69**, 1241–1251. 10.1107/S0907444913003880 PMC3689527 23793150

[bb13] Casanas, A., Warshamanage, R., Finke, A. D., Panepucci, E., Olieric, V., Nöll, A., Tampé, R., Brandstetter, S., Förster, A., Mueller, M., Schulze-Briese, C., Bunk, O. & Wang, M. (2016). *Acta Cryst.* D**72**, 1036–1048. 10.1107/S2059798316012304 PMC5013597 27599736

[bb14] Chapman, H. N., Fromme, P., Barty, A., White, T. A., Kirian, R. A., Aquila, A., Hunter, M. S., Schulz, J., DePonte, D. P., Weierstall, U., Doak, R. B., Maia, F. R. N. C., Martin, A. V., Schlichting, I., Lomb, L., Coppola, N., Shoeman, R. L., Epp, S. W., Hartmann, R., Rolles, D., Rudenko, A., Foucar, L., Kimmel, N., Weidenspointner, G., Holl, P., Liang, M., Barthelmess, M., Caleman, C., Boutet, S., Bogan, M. J., Krzywinski, J., Bostedt, C., Bajt, S., Gumprecht, L., Rudek, B., Erk, B., Schmidt, C., Hömke, A., Reich, C., Pietschner, D., Strüder, L., Hauser, G., Gorke, H., Ullrich, J., Herrmann, S., Schaller, G., Schopper, F., Soltau, H., Kühnel, K., Messerschmidt, M., Bozek, J. D., Hau-Riege, S. P., Frank, M., Hampton, C. Y., Sierra, R. G., Starodub, D., Williams, G. J., Hajdu, J., Timneanu, N., Seibert, M. M., Andreasson, J., Rocker, A., Jönsson, O., Svenda, M., Stern, S., Nass, K., Andritschke, R., Schröter, C., Krasniqi, F., Bott, M., Schmidt, K. E., Wang, X., Grotjohann, I., Holton, J. M., Barends, T. R. M., Neutze, R., Marchesini, S., Fromme, R., Schorb, S., Rupp, D., Adolph, M., Gorkhover, T., Andersson, I., Hirsemann, H., Potdevin, G., Graafsma, H., Nilsson, B. & Spence, J. C. H. (2011). *Nature*, **470**, 73–77.

[bb15] Cipriani, F., Röwer, M., Landret, C., Zander, U., Felisaz, F. & Márquez, J. A. (2012). *Acta Cryst.* D**68**, 1393–1399. 10.1107/S0907444912031459 22993093

[bb16] Cornaciu, I., Bourgeas, R., Hoffmann, G., Dupeux, F., Humm, A.-S., Mariaule, V., Pica, A., Clavel, D., Seroul, G., Murphy, P. & Márquez, J. A. (2021). *J. Vis. Exp.* **172**, e62491. 10.3791/62491 34152315

[bb17] Delagenière, S., Brenchereau, P., Launer, L., Ashton, A. W., Leal, R., Veyrier, S., Gabadinho, J., Gordon, E. J., Jones, S. D., Levik, K. E., McSweeney, S. M., Monaco, S., Nanao, M., Spruce, D., Svensson, O., Walsh, M. A. & Leonard, G. A. (2011). *Bioinformatics*, **27**, 3186–3192. 10.1093/bioinformatics/btr535 21949273

[bb18] DeLano, W. L. (2002). *CCP4 Newsl. Protein Crystallogr.* **40**, 82–92.

[bb19] De Wijn, R., Rollet, K., Olieric, V., Hennig, O., Thome, N., Noûs, C., Paulus, C., Lorber, B., Betat, H., Mörl, M. & Sauter, C. (2021). *J. Vis. Exp.* pp. e61972. 10.3791/61972 33818565

[bb20] Diederichs, K. & Wang, M. (2017). *Protein Crystallography*, Vol. 1607, edited by A. Wlodawer, Z. Dauter & M. Jaskolski, pp. 239–272. New York: Springer.

[bb21] Doukov, T., Herschlag, D. & Yabukarski, F. (2020). *J. Appl. Cryst.* **53**, 1493–1501. 10.1107/S1600576720013503 PMC7710493 33312102

[bb22] Duret, L., Gasteiger, E. & Perrièe, G. (1996). *Bioinformatics*, **12**, 507–510. 10.1093/bioinformatics/12.6.507 9021269

[bb23] Emsley, P., Lohkamp, B., Scott, W. G. & Cowtan, K. (2010). *Acta Cryst.* D**66**, 486–501. 10.1107/S0907444910007493 PMC2852313 20383002

[bb24] Evans, P. R. & Murshudov, G. N. (2013). *Acta Cryst.* D**69**, 1204–1214. 10.1107/S0907444913000061 PMC3689523 23793146

[bb25] Eymery, M. C., McCarthy, A. A. & Hausmann, J. (2023). *Life Sci. Alliance*, **6**, e202201595. 10.26508/lsa.202201595 PMC9834664 36623871

[bb26] Eymery, M. C., Nguyen, K.-A., Basu, S., Hausmann, J., Tran-Nguyen, V.-K., Seidel, H. P., Gutierrez, L., Boumendjel, A. & McCarthy, A. A. (2024). *Eur. J. Med. Chem.* **263**, 115944. 10.1016/j.ejmech.2023.115944 37976710

[bb27] Fischer, M. (2021). *Q. Rev. Biophys.* **54**, e1. 10.1017/S0033583520000128 33413726

[bb28] Fraser, J. S., van den Bedem, H., Samelson, A. J., Lang, P. T., Holton, J. M., Echols, N. & Alber, T. (2011). *Proc. Natl Acad. Sci. USA*, **108**, 16247–16252. 10.1073/pnas.1111325108 PMC3182744 21918110

[bb29] French, S. & Wilson, K. (1978). *Acta Cryst.* A**34**, 517–525.

[bb30] Gavira, J. A., Rodriguez-Ruiz, I., Martinez-Rodriguez, S., Basu, S., Teychené, S., McCarthy, A. A. & Mueller-Dieckman, C. (2020). *Acta Cryst.* D**76**, 751–758. 10.1107/S2059798320008475 32744257

[bb31] Gevorkov, Y., Yefanov, O., Barty, A., White, T. A., Mariani, V., Brehm, W., Tolstikova, A., Grigat, R.-R. & Chapman, H. N. (2019). *Acta Cryst.* A**75**, 694–704. 10.1107/S2053273319010593 PMC6718201 31475914

[bb32] J. Gildea, R., Orr, C. M., Paterson, N. G. & Hall, D. R. (2022). *Synchrotron Radiat. News* **35**, 51–54.

[bb33] Hausmann, J., Kamtekar, S., Christodoulou, E., Day, J. E., Wu, T., Fulkerson, Z., Albers, H. M. H. G., van Meeteren, L. A., Houben, A. J. S., van Zeijl, L., Jansen, S., Andries, M., Hall, T., Pegg, L. E., Benson, T. E., Kasiem, M., Harlos, K., Kooi, C. W. V., Smyth, S. S., Ovaa, H., Bollen, M., Morris, A. J., Moolenaar, W. H. & Perrakis, A. (2011). *Nat. Struct. Mol. Biol.* **18**, 198–204. 10.1038/nsmb.1980 PMC3064516 21240271

[bb34] Healey, R. D., Basu, S., Humm, A.-S., Leyrat, C., Cong, X., Golebiowski, J., Dupeux, F., Pica, A., Granier, S. & Márquez, J. A. (2021). *Cell Rep. Methods*, **1**, 100102. 10.1016/j.crmeth.2021.100102 PMC8545655 34723237

[bb35] Homs, A., Claustre, L., Kirov, A., Papillon, E. & Petitdemange, S. (2011). *Proceedings of the 13th International Conference on Accelerators and Large Experimental Physics Control Systems (ICALEPCS2011)*, 10–14 October 2011, Grenoble, France, pp. 676–679. Geneva: JaCOW.

[bb37] Huang, C., Olieric, V., Howe, N., Warshamanage, R., Weinert, T., Panepucci, E., Vogeley, L., Basu, S., Diederichs, K., Caffrey, M. & Wang, M. (2018). *Commun. Biol.* **1**, 124. 10.1038/s42003-018-0123-6 PMC6123769 30272004

[bb36] Huang, C.-Y., Aumonier, S., Engilberge, S., Eris, D., Smith, K. M. L., Leonarski, F., Wojdyla, J. A., Beale, J. H., Buntschu, D., Pauluhn, A., Sharpe, M. E., Metz, A., Olieric, V. & Wang, M. (2022). *Acta Cryst.* D**78**, 964–974. 10.1107/S205979832200612X PMC9344481 35916221

[bb38] Huang, C.-Y., Olieric, V., Ma, P., Panepucci, E., Diederichs, K., Wang, M. & Caffrey, M. (2015). *Acta Cryst.* D**71**, 1238–1256. 10.1107/S1399004715005210 PMC4461204 26057665

[bb39] Kabsch, W. (2010). *Acta Cryst.* D**66**, 125–132. 10.1107/S0907444909047337 PMC2815665 20124692

[bb40] Karplus, P. A. & Diederichs, K. (2012). *Science*, **336**, 1030–1033. 10.1126/science.1218231 PMC3457925 22628654

[bb41] Keedy, D. A., Hill, Z. B., Biel, J. T., Kang, E., Rettenmaier, T. J., Brandão-Neto, J., Pearce, N. M., von Delft, F., Wells, J. A. & Fraser, J. S. (2018). *eLife*, **7**, e36307. 10.7554/eLife.36307 PMC6039181 29877794

[bb42] Keedy, D. A., Kenner, L. R., Warkentin, M., Woldeyes, R. A., Hopkins, J. B., Thompson, M. C., Brewster, A. S., Van Benschoten, A. H., Baxter, E. L., Uervirojnangkoorn, M., McPhillips, S. E., Song, J., Alonso-Mori, R., Holton, J. M., Weis, W. I., Brunger, A. T., Soltis, S. M., Lemke, H., Gonzalez, A., Sauter, N. K., Cohen, A. E., van den Bedem, H., Thorne, R. E. & Fraser, J. S. (2015). *eLife*, **4**, e07574. 10.7554/eLife.07574 PMC4721965 26422513

[bb43] Kühlbrandt, W. (2014). *Science*, **343**, 1443–1444. 10.1126/science.1251652 24675944

[bb44] Maire, A. le, Gelin, M., Pochet, S., Hoh, F., Pirocchi, M., Guichou, J.-F., Ferrer, J.-L. & Labesse, G. (2011). *Acta Cryst.* D**67**, 747–755. 10.1107/S0907444911023249 21904027

[bb45] Márquez, J. A. & Cipriani, F. (2014). *Methods Mol. Biol.* **1091**, 197–203. 10.1007/978-1-62703-691-7_14 24203334

[bb46] McCarthy, A. A., Barrett, R., Beteva, A., Caserotto, H., Dobias, F., Felisaz, F., Giraud, T., Guijarro, M., Janocha, R., Khadrouche, A., Lentini, M., Leonard, G. A., Lopez Marrero, M., Malbet-Monaco, S., McSweeney, S., Nurizzo, D., Papp, G., Rossi, C., Sinoir, J., Sorez, C., Surr, J., Svensson, O., Zander, U., Cipriani, F., Theveneau, P. & Mueller-Dieckmann, C. (2018). *J. Synchrotron Rad.* **25**, 1249–1260. 10.1107/S1600577518007166 PMC6038607 29979188

[bb47] McCoy, A. J. (2007). *Acta Cryst.* D**63**, 32–41. 10.1107/S0907444906045975 PMC2483468 17164524

[bb48] Mikolajek, H., Sanchez-Weatherby, J., Sandy, J., Gildea, R. J., Campeotto, I., Cheruvara, H., Clarke, J. D., Foster, T., Fujii, S., Paulsen, I. T., Shah, B. S. & Hough, M. A. (2023). *IUCrJ*, **10**, 420–429. 10.1107/S2052252523003810 PMC10324489 37199504

[bb49] Moolenaar, W. H. & Perrakis, A. (2011). *Nat. Rev. Mol. Cell Biol.* **12**, 674–679. 10.1038/nrm3188 21915140

[bb50] Moreno-Chicano, T., Ebrahim, A., Axford, D., Appleby, M. V., Beale, J. H., Chaplin, A. K., Duyvesteyn, H. M. E., Ghiladi, R. A., Owada, S., Sherrell, D. A., Strange, R. W., Sugimoto, H., Tono, K., Worrall, J. A. R., Owen, R. L. & Hough, M. A. (2019). *IUCrJ*, **6**, 1074–1085. 10.1107/S2052252519011655 PMC6830213 31709063

[bb51] Morin, A., Eisenbraun, B., Key, J., Sanschagrin, P. C., Timony, M. A., Ottaviano, M. & Sliz, P. (2013). *eLife*, **2**, e01456. 10.7554/eLife.01456 PMC3771563 24040512

[bb52] Nanao, M., Basu, S., Zander, U., Giraud, T., Surr, J., Guijarro, M., Lentini, M., Felisaz, F., Sinoir, J., Morawe, C., Vivo, A., Beteva, A., Oscarsson, M., Caserotto, H., Dobias, F., Flot, D., Nurizzo, D., Gigmes, J., Foos, N., Siebrecht, R., Roth, T., Theveneau, P., Svensson, O., Papp, G., Lavault, B., Cipriani, F., Barrett, R., Clavel, C. & Leonard, G. (2022). *J. Synchrotron Rad.* **29**, 581–590.

[bb53] Oscarsson, M., Beteva, A., Flot, D., Gordon, E., Guijarro, M., Leonard, G., McSweeney, S., Monaco, S., Mueller-Dieckmann, C., Nanao, M., Nurizzo, D., Popov, A., von Stetten, D., Svensson, O., Rey-Bakaikoa, V., Chado, I., Chavas, L., Gadea, L., Gourhant, P., Isabet, T., Legrand, P., Savko, M., Sirigu, S., Shepard, W., Thompson, A., Mueller, U., Nan, J., Eguiraun, M., Bolmsten, F., Nardella, A., Milàn-Otero, A., Thunnissen, M., Hellmig, M., Kastner, A., Schmuckermaier, L., Gerlach, M., Feiler, C., Weiss, M. S., Bowler, M. W., Gobbo, A., Papp, G., Sinoir, J., McCarthy, A., Karpics, I., Nikolova, M., Bourenkov, G., Schneider, T., Andreu, J., Cuní, G., Juanhuix, J., Boer, R., Fogh, R., Keller, P., Flensburg, C., Paciorek, W., Vonrhein, C., Bricogne, G. & de Sanctis, D. (2019). *J. Synchrotron Rad.* **26**, 393–405. 10.1107/S1600577519001267 PMC6412183 30855248

[bb54] Pearson, A. R. & Mehrabi, P. (2020). *Curr. Opin. Struct. Biol.* **65**, 168–174. 10.1016/j.sbi.2020.06.019 32846363

[bb55] Raimondi, P., Benabderrahmane, C., Berkvens, P., Biasci, J. C., Borowiec, P., Bouteille, J.-F., Brochard, T., Brookes, N. B., Carmignani, N., Carver, L. R., Chaize, J.-M., Chavanne, J., Checchia, S., Chushkin, Y., Cianciosi, F., Di Michiel, M., Dimper, R., D’Elia, A., Einfeld, D., Ewald, F., Farvacque, L., Goirand, L., Hardy, L., Jacob, J., Jolly, L., Krisch, M., Le Bec, G., Leconte, I., Liuzzo, S. M., Maccarrone, C., Marchial, T., Martin, D., Mezouar, M., Nevo, C., Perron, T., Plouviez, E., Reichert, H., Renaud, P., Revol, J.-L., Roche, B., Scheidt, K.-B., Serriere, V., Sette, F., Susini, J., Torino, L., Versteegen, R., White, S. & Zontone, F. (2023). *Commun. Phys.* **6**, 82.

[bb56] Read, R. J. (2001). *Acta Cryst.* D**57**, 1373–1382. 10.1107/s0907444901012471 11567148

[bb57] Russi, S., González, A., Kenner, L. R., Keedy, D. A., Fraser, J. S. & van den Bedem, H. (2017). *J. Synchrotron Rad.* **24**, 73–82. 10.1107/S1600577516017343 PMC5182021 28009548

[bb58] Stein, A. J., Bain, G., Prodanovich, P., Santini, A. M., Darlington, J., Stelzer, N. M. P., Sidhu, R. S., Schaub, J., Goulet, L., Lonergan, D., Calderon, I., Evans, J. F. & Hutchinson, J. H. (2015). *Mol. Pharmacol.* **88**, 982–992. 10.1124/mol.115.100404 26371182

[bb59] Stellato, F., Oberthür, D., Liang, M., Bean, R., Gati, C., Yefanov, O., Barty, A., Burkhardt, A., Fischer, P., Galli, L., Kirian, R. A., Meyer, J., Panneerselvam, S., Yoon, C. H., Chervinskii, F., Speller, E., White, T. A., Betzel, C., Meents, A. & Chapman, H. N. (2014). *IUCrJ*, **1**, 204–212. 10.1107/S2052252514010070 PMC4107920 25075341

[bb60] Thompson, A. J., Sanchez-Weatherby, J., Williams, L. J., Mikolajek, H., Sandy, J., Worrall, J. A. R. & Hough, M. A. (2024). *Acta Cryst.* D**80**, 279–288. 10.1107/S2059798324001955 PMC10994175 38488731

[bb61] Weinert, T., Olieric, N., Cheng, R., Brünle, S., James, D., Ozerov, D., Gashi, D., Vera, L., Marsh, M., Jaeger, K., Dworkowski, F., Panepucci, E., Basu, S., Skopintsev, P., Doré, A. S., Geng, T., Cooke, R. M., Liang, M., Prota, A. E., Panneels, V., Nogly, P., Ermler, U., Schertler, G., Hennig, M., Steinmetz, M. O., Wang, M. & Standfuss, J. (2017). *Nat Commun*, **8**, 542. 10.1038/s41467-017-00630-4 PMC5599499 28912485

[bb62] White, T. A., Kirian, R. A., Martin, A. V., Aquila, A., Nass, K., Barty, A. & Chapman, H. N. (2012). *J. Appl. Cryst.* **45**, 335–341.

[bb63] Williams, C. J., Headd, J. J., Moriarty, N. W., Prisant, M. G., Videau, L. L., Deis, L. N., Verma, V., Keedy, D. A., Hintze, B. J., Chen, V. B., Jain, S., Lewis, S. M., Arendall, W. B., Snoeyink, J., Adams, P. D., Lovell, S. C., Richardson, J. S. & Richardson, D. C. (2018). *Protein Sci.* **27**, 293–315. 10.1002/pro.3330 PMC5734394 29067766

[bb64] Wlodawer, A., Minor, W., Dauter, Z. & Jaskolski, M. (2008). *FEBS J.* **275**, 1–21. 10.1111/j.1742-4658.2007.06178.x PMC4465431 18034855

[bb65] Zander, U., Bourenkov, G., Popov, A. N., de Sanctis, D., Svensson, O., McCarthy, A. A., Round, E., Gordeliy, V., Mueller-Dieckmann, C. & Leonard, G. A. (2015). *Acta Cryst.* D**71**, 2328–2343. 10.1107/S1399004715017927 PMC4631482 26527148

[bb66] Zeldin, O. B., Brockhauser, S., Bremridge, J., Holton, J. M. & Garman, E. F. (2013). *Proc. Natl Acad. Sci. USA*, **110**, 20551–20556. 10.1073/pnas.1315879110 PMC3870734 24297937

