# Supplementary material for: In situ serial crystallography facilitates 96-well plate structural analysis at low symmetry
Source: IUCrJ. 2024 Jul 15;11(Pt 5):780–91. doi: 10.1107/S2052252524005785 (PMC11364034; doi:10.1107/S2052252524005785)
Supplement: Supplementary file 1 [file m-11-00780-sup1.pdf]

IUCrJ

Volume 11 (2024)

Supporting information for article:

In situ serial crystallography facilitates 96-well plate for structural analysis at low symmetry

Nicolas Foos, Jean-Baptise Florial, Mathias Eymery, Jeremy Sinoir, Franck Felisaz, Marcus Oscarsson, Antonia Beteva, Matthew W. Bowler, Didier Nurizzo, Gergely Papp, Montserrat Soler-Lopez, Max Nanao, Shibom Basu and Andrew A. McCarthy

S1. ***MXCuBE-Web*** for iSX data collection

The motorised crystal plate manipulator (PM) has been integrated into *MXCuBE-Web* as a new hardware object, which is called when PM is the goniometer-head. The hardware-object for PM is compatible with MD2S and MD3-Up diffractometers.

The PM hardware-object is based on Sample Changer object (or Class), exploiting all the sample changer characteristic such as: a *cell* in the sample-changer Dewar is substituted with a *row* in the 96 well-plate, a well in the plate represents a puck in the sample-changer Dewar, and a drop in the well represents the sample. It also has the mounting sample functionality, which implies a plate translational motion (horizontal and vertical) to position a drop. A plate class is organised in rows and columns – each well (Cell object) contains a drop (Drop object) and each drop could contain several crystals (Xtal object).

The PM hardware object is implemented in both *QT* and the latest Web versions of the *MXCuBE*. In the case of *MXCuBE-2*, based on *PyQt4*, the graphical user interface (GUI) was created by combining self-contained widgets. In the *MXCuBE-Web* version, the GUI is implemented with Javascript/ React-17 with the React-Bootstrap and the react-contexify libraries, displaying a more elegant context menu. In both versions, the GUI representing the 96-well plate provides a mode of interaction with the hardware object that controls the physical instrument. The code for *MXCuBE-Web* is open-source and freely available at https://github.com/mxcube/mxcubeweb. As a cross-facilities collaborative project, *MXCuBE-Web* and its plate manipulator implementation is available to other synchrotron beamlines.

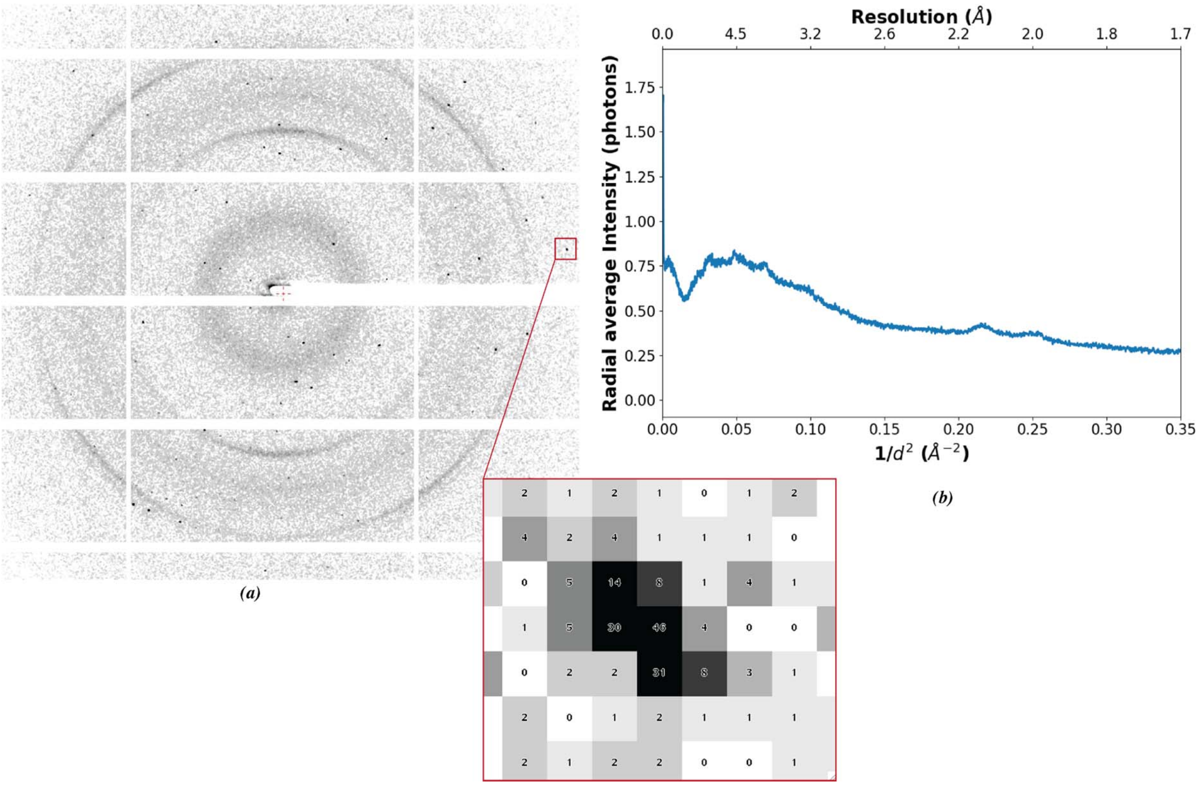

Figure S1 Diffraction quality and background estimation. *(a)* An example diffraction pattern from iSX dataset. A high-resolution spot on the detector edge is highlighted in a red box and spot intensity together with surrounding background level has been shown in the zoomed inset at the bottom of the panel. *(b)* 1D plot shows the radially average background estimation as a function of resolution for the top (Polyolefin ultra-thin film, HJ Bioanalytik, GmbH) and bottom layers (ultra-thin COC-based film) of the CrystalDirect plate together with crystallisation drop at the equivalent X-ray flux.

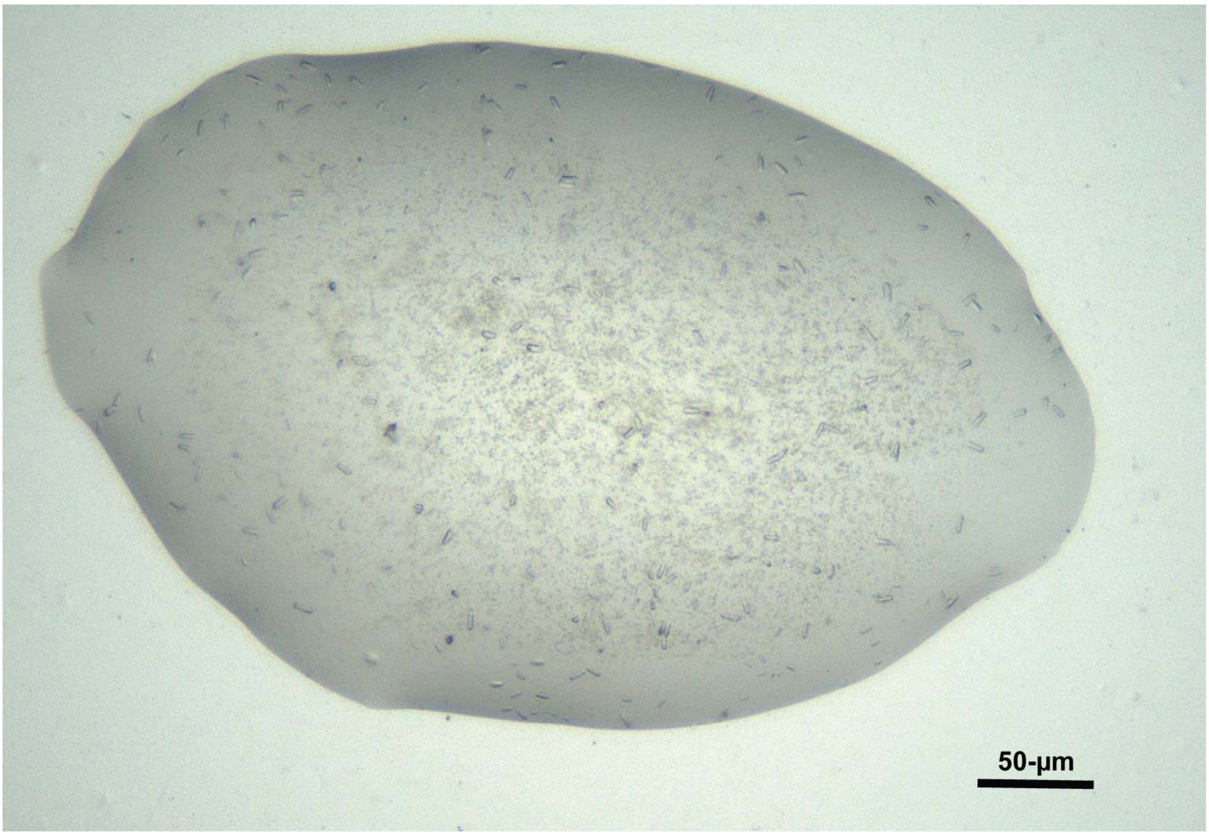

Figure S2 Visible microscope image of an example crystallisation drop depicting the microcrystals of r-ATX. The microcrystals are typically 5 x 5 x 10 μm^3 in size.

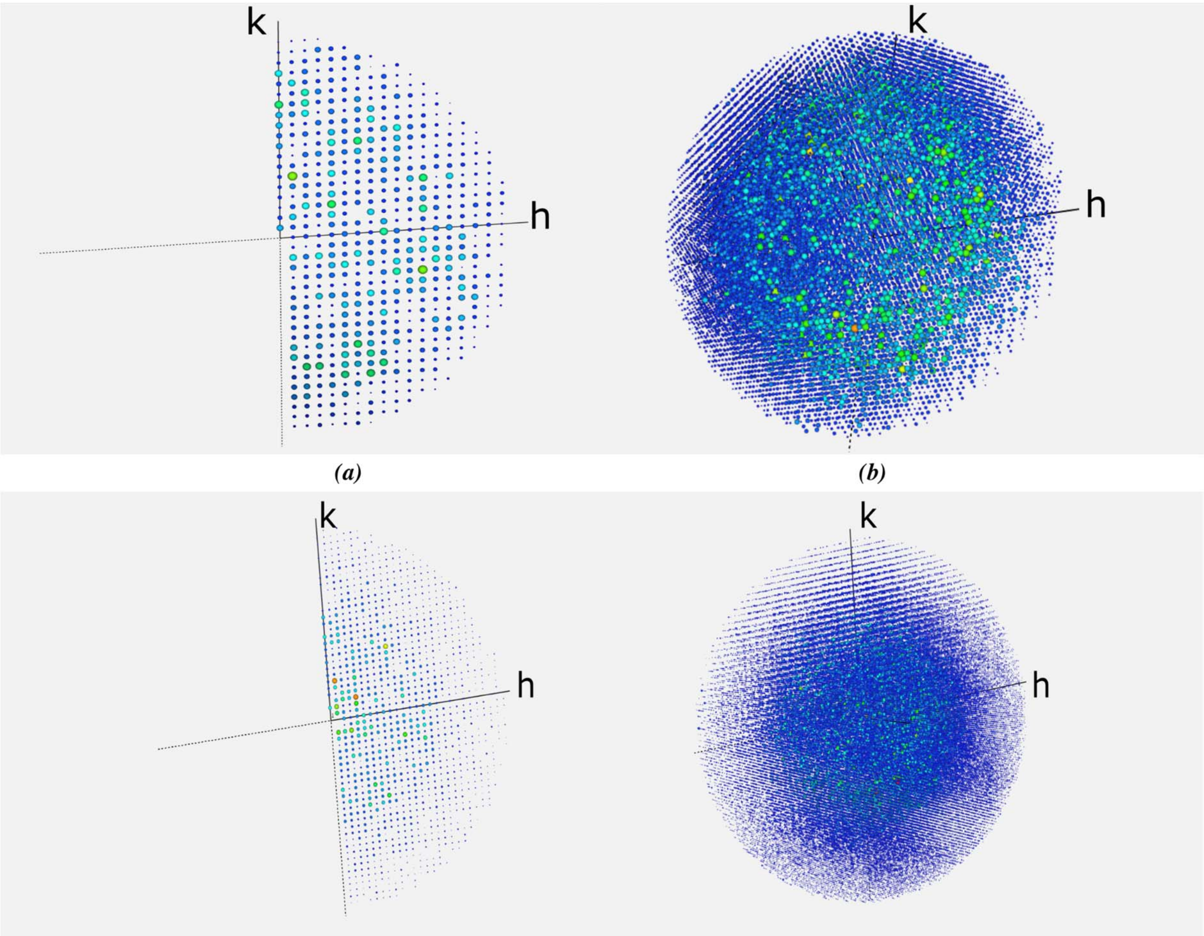

(c) *(d)*

Figure S3 HKL-view in 2D and 3D for iSX and cryo-SSX datasets. Rainbow coloured 2D representation of Bragg reflections from the iSX and cryo-SSX datasets are shown as projections along the *l* axis on the *h*-*k* plan in panels *(a)* and *(c)*, respectively. 3D rainbow coloured representation of Bragg reflections from the iSX and cryo-SSX datasets in panels *(b)* and *(d)*, respectively, showing a complete dataset with a full sampling of the reciprocal space. The resolution cut for the representation is 3Å.

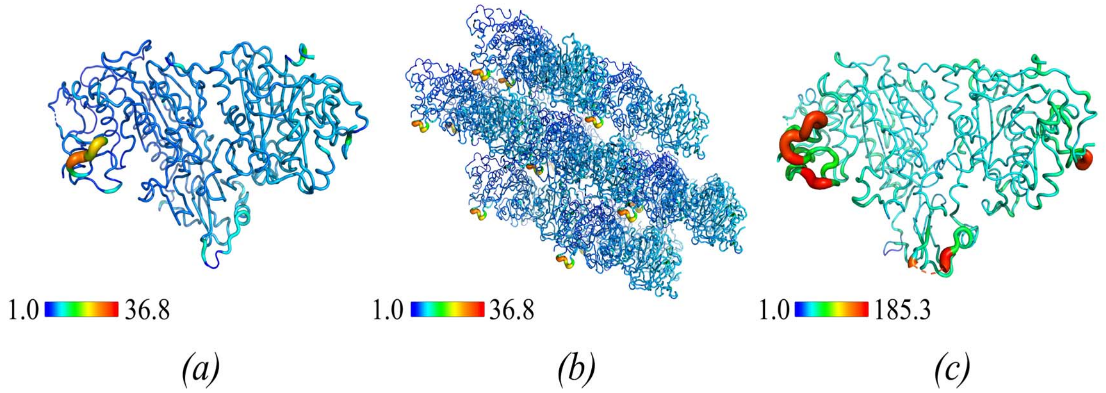

Figure S4 RMSD (Å) between r-ATX-RT and r-ATX-Cryo structures *(a)* and *(b)*, and comparison of B-factors (Å^2) between r-ATX-RT and r-ATX-Cryo structures *(c)*. *(a)* Putty representation of ATX-RT model rainbow coloured. Colour and tube diameter representing the Cα RMSD (Å) value calculated between ATX-RT and ATX-Cryo. Loops with higher B-factor difference are shown in red and large tube diameter, and those with the lower B-factor difference are in dark blue with reduced tube diameter. *(b)* Crystal packing view. *(c)* Putty representation of the r-ATX-RT model rainbow coloured. Colour and tube diameter representing the Cα B-factors (Å^2) differences between r-ATX-RT and r-ATX-Cryo. Loops for which the B-factor difference are higher are represented in red and large tube diameter, and those with the lower difference are in blue with reduced tube diameter.
